# Artificial Enamel

**Published:** 1909-06-15

**Authors:** Harry L. Belcher

**Affiliations:** Seneca Falls, N. Y.


### "ARTIFICIAL ENAMEL."

BY HARRY L. BELCHER, D.D.S., SENECA FALLS, N. Y.

Read before Rochester Dental Society.

Of the silicate cements, my experience for the past three years or more has been confined to "Aschers Artificial Enamel." My remarks, therefore, will be confined to this material. In conversation with dentists a number have said that they did not believe they knew how to mix this material. In reading the directions, many of us seem to have a different conception of how the thing should be done, and perhaps do not have sufficient respect or faith in what the manufacturer says, because of our many years of practice. We are dealing with "a chemical porcelain" and with which the average dentist is not familiar, nor has he the time to study chemical reaction. One of my confreres had been very enthusiastic about this material, but, to my surprise, in a few weeks he abandoned it. He said "The stuff is no good." I asked him how he mixed it and he said "The same as you would any other cement."

Surely one cannot get satisfactory results, unless there is exactness in the mixing.

The "mix" should contain as much of the body as possible and the mass should be perfected as quickly as possible and inserted into the cavity before chemical action ceases. After the "enamel" has once started to set, manipulation should cease, for if the crystals are broken, the structure will be weakened. You have about two minutes before the mix commences to set. Still further in regard to the detail of mixing, the manufacturers say, that the powder should be gradually worked into the liquid, with comparatively little spatulation; in other words, we should avoid *pressure* with the spatula in the mixing—constant heavy pressure breaks up the crystals. The last few strokes of the spatula, after the paste has become tough, is pressure that may be safely used. Another important point is to continuously add the powder in small quantities, using only a very light motion with the spatula. Let the liquid take up all the powder it will, without thoroughly spatulating. Spatulating in this case is misleading. The word "spatulate" means to spread out: and that is what we should avoid, that is, not to spread the material any more than is necessary.

Use the smallest size spatula with which you can handle the mass. It is important to make a proper mix by using a very large spatula. The liquid spreads over the slab, and a large spatula makes it impossible to work sufficient powder into the liquid unless an unnecessarily large quantity of the latter is used. A stiffer mix may be used in small, easily reached cavities, than can be properly inserted in large compound cavities having extensive margins, but the stiffness should be due to the larger proportion of powder used, rather than delay from any cause even prolonged mixing.

The shade should be selected before the rubber dam is adjusted, as the shade of the tooth is altered during cavity preparation with the rubber dam on. It is best to choose a shade darker than the tooth: one is apt to get the shade too light. Regarding the use of two or more shades, there is no objection if there is not too much delay in mixing the material. If you can obtain the desired result with one shade it is more advisable, as you can count on a more definite and satisfactory result each time. When the shade is correct, you have something that is very gratifying to patient and operator alike, because of the translucency and tooth-like appearance of the material and a joint that is almost invisible. The question of shade is more satisfactory with silicate cements than with the fused porcelain. The silicate is more homogenous throughout and transmits, to a degree, the color of the tooth; while with a porcelain inlay, perfect in shade before setting, this is entirely changed when set—due to the stratum of opaque cement underneath the porcelain.

My first experiences with this material were quite unsatisfactory at times, and I had more or less trouble with discoloration of the filling after it had been placed. I was inclined to blame the material but, as I look back, I am convinced it was due to my own inability; those same troubles have not occurred in the last two years practically.

One of the first causes of discoloration is the excessive use of vaseline. Vaseline is not supposed to be combined with this material; even in small amounts it will surely effect discoloration. The only thing on which we have any license to use vaseline is the celluloid strip in finishing, and then only as a very slight coating. If the material sticks to the instrument, this is at once twisted in a clean napkin and then quickly dipped in pure grain alcohol—this eliminates the trouble at once. The instruments may be frequently dipped in alcohol; the amount of alcohol used is infinitesimal and does not injure the surface of the material in any way.

Another cause of discoloration is the bone spatula, which has been carelessly allowed to become water soaked; this puts it in nice shape to take up dirt and septic matter. Other causes are the use of one slab for all kinds of cements, the use of metal instruments, perhaps used in amalgam work; careless use of strips, disks and stones, that sometimes carry material from clamps and metal fillings. New and clean corundum and Arkansas stones should be kept exclusively for this work, in case it is necessary to use them.

There is no material that requires more exacting attention to minute detail than does the silicate cements; therefore, do not place this material in a patient's mouth, until you understand clearly what is set forth in the directions by the manufacturer. This material can be safely employed in any place where you would put a gold filling—*where there is no stress.*

The rubber dam should be used in every case. If you cannot place the dam the conditions can not be made right and, in the name of conservative dentistry, employ some other material than silicate cement.

